# Design and implementation of the START (STem cells for ARDS Treatment) trial, a phase 1/2 trial of human mesenchymal stem/stromal cells for the treatment of moderate-severe acute respiratory distress syndrome

**DOI:** 10.1186/s13613-014-0022-z

**Published:** 2014-07-03

**Authors:** Kathleen D Liu, Jennifer G Wilson, Hanjing Zhuo, Lizette Caballero, Melanie L McMillan, Xiaohui Fang, Katherine Cosgrove, Carolyn S Calfee, Jae-Woo Lee, Kirsten N Kangelaris, Jeffrey E Gotts, Angela J Rogers, Joseph E Levitt, Jeanine P Wiener-Kronish, Kevin L Delucchi, Andrew D Leavitt, David H McKenna, B Taylor Thompson, Michael A Matthay

**Affiliations:** 1 Departments of Nephrology and Anesthesia, University of California, San Francisco, CA, USA; 2 Departments of Emergency Medicine and Critical Care Medicine, University of California, San Francisco, CA, USA; 3 Cardiovascular Research Institute, University of California, San Francisco, CA, USA; 4 Clinical Laboratories, Bone Marrow Center, University of California, San Francisco, CA, USA; 5 Division of Pulmonary and Critical Care Medicine, Department of Medicine, Massachusetts General Hospital, Boston, MA, USA; 6 Division of Pulmonary and Critical Care Medicine, Department of Medicine, University of California, San Francisco, CA, USA; 7 Department of Anesthesia, University of California, San Francisco, CA, USA; 8 Division of Hospital Medicine, Department of Medicine, University of California, San Francisco, CA, USA; 9 Division of Pulmonary and Critical Care Medicine, Department of Medicine, Stanford University, Stanford, CA, USA; 10 Department of Anesthesia, Critical Care and Pain Medicine, Massachusetts General Hospital, Harvard University, Cambridge, MA, USA; 11 Department of Psychiatry, University of California, San Francisco, CA, USA; 12 Department of Laboratory Medicine, University of California, San Francisco, CA, USA; 13 Division of Transfusion Medicine, Department of Laboratory Medicine and Pathology, University of Minnesota, Minneapolis, MN, USA; 14 Departments of Medicine and Anesthesia and the Cardiovascular Research Institute, University of California, San Francisco, CA, USA

**Keywords:** Acute lung injury, Clinical trial, Mesenchymal stem/stromal cell, Pulmonary edema

## Abstract

**Background:**

Despite advances in supportive care, moderate-severe acute respiratory distress syndrome (ARDS) is associated with high mortality rates, and novel therapies to treat this condition are needed. Compelling pre-clinical data from mouse, rat, sheep and ex vivo perfused human lung models support the use of human mesenchymal stem (stromal) cells (MSCs) as a novel intravenous therapy for the early treatment of ARDS.

**Methods:**

This article describes the study design and challenges encountered during the implementation and phase 1 component of the START (STem cells for ARDS Treatment) trial, a phase 1/2 trial of bone marrow-derived human MSCs for moderate-severe ARDS. A trial enrolling 69 subjects is planned (9 subjects in phase 1, 60 subjects in phase 2 treated with MSCs or placebo in a 2:1 ratio).

**Results:**

This report describes study design features that are unique to a phase 1 trial in critically ill subjects and the specific challenges of implementation of a cell-based therapy trial in the ICU.

**Conclusions:**

Experience gained during the design and implementation of the START study will be useful to investigators planning future phase 1 clinical trials based in the ICU, as well as trials of cell-based therapy for other acute illnesses.

**Trial registration:**

Clinical Trials Registration: NCT01775774 and NCT02097641.

## Background

Morbidity and mortality have declined only modestly in patients with the acute respiratory distress syndrome (ARDS) in the last decade despite extensive research into its pathophysiology [1]-[3]. Current treatment remains primarily supportive with lung-protective ventilation and a fluid conservative strategy [4],[5]. Pharmacologic therapies that reduce the severity of lung injury *in vivo* and *in vitro* have not yet been translated to effective clinical treatment options. At present, the mortality rate of severe ARDS remains unacceptably high, in the range of 30 to 40% [6],[7]. Therefore, innovative therapies are needed, in particular for individuals with moderate-severe ARDS who have the highest mortality rates.

Cell-based therapy with human mesenchymal stem/stromal cells (MSCs) is attractive as a potential new treatment for ARDS for multiple reasons. MSCs have the ability to secrete multiple paracrine factors, such as growth factors that can enhance tissue repair, anti-inflammatory cytokines that can reduce inflammation, and antimicrobial peptides that can reduce the severity of bacterial infection [8]-[17]. MSCs can also transfer mitochondria to injured lung epithelial cells, thus enhancing their functional status by replenishing depleted levels of ATP [18]. All of these mechanisms are relevant to the major abnormalities that underlie ARDS, including impaired alveolar fluid clearance, altered lung endothelial and epithelial permeability, dysregulated inflammation and ongoing infection. To date, MSCs have been tested for a variety of indications in more than 2,000 human subjects as therapy for a variety of diseases due to their low immunogenicity, immunomodulatory effects, and ability to secrete endothelial and epithelial growth factors.

Pre-clinical studies in small and large animal models (mouse, rat, and sheep), as well as in an *ex vivo* perfused human lung model, have demonstrated the potential efficacy and safety of administering human MSCs for the treatment of ARDS [8],[9],[11],[12],[14]. Based on these studies, we proposed a phase 1 dose escalation trial followed by a phase 2 randomized, double-blind, placebo-controlled clinical trial of human MSCs for the treatment of moderate-severe ARDS. The purpose of this article is to describe the design of this clinical trial and the particular challenges we faced in testing a cell-based therapy in a critically ill population of patients with moderate-severe ARDS. We also present practical lessons learned during the implementation of the phase 1 trial relevant to both future trials of cell-based therapies and to other phase 1 trials in critically ill patients.

## Methods

### Study overview

The START trial is a multicenter phase 1/phase 2 clinical trial of MSCs in patients with moderate-severe ARDS, defined as a PaO_2_/FiO_2_ ratio of less than 200 mmHg while on positive pressure ventilation with at least 8 cmH_2_O positive end-expiratory pressure (PEEP) [19].

The phase 1 component of the study is an open label dose escalation pilot study in which three cohorts of subjects with ARDS receive increasing doses of human MSCs administered as a single intravenous infusion. There are three subjects per cohort, with patients in each cohort receiving either 1 × 10^6^ cells/kg predicted body weight (first cohort), 5 × 10^6^ cells/kg predicted body weight (second cohort), or 10 × 10^6^ cells/kg predicted body weight (third cohort).

Phase 2 is a randomized, double-blind placebo-controlled study using the maximally tolerated dose (MTD) of cells from the phase 1 study (up to 10 × 10^6^ cell/kg predicted body weight). The MTD is the highest dose that is associated with no pre-specified infusion associated events or unexpected severe adverse attributed to the study product. Subjects will be randomized in a 2:1 randomization scheme to receive human MSCs or Plasma-lyte A placebo; the study will enroll 60 patients who achieve a stable clinical baseline and receive the investigational product. The coordinating center for the trial is at the University of California, San Francisco. Eligible study subjects will be enrolled in the phase 1 trial at the University of California, San Francisco, Stanford University, and the Massachusetts General Hospital. Planned phase 2 sites also include the University of Pittsburgh and the University of Vermont. The trial is funded by the National Institutes of Health through a Clinical and Translational Science Institute award to the University of California, San Francisco and through the National Heart, Lung and Blood Institute (NHLBI) Pilot Trials in Lung Disease U01 mechanism and the NHLBI-supported Production Assistance for Cellular Therapies (PACT) program at the University of Minnesota. The human MSCs are prepared from donor bone marrow at the University of Minnesota and shipped frozen to the clinical sites. Prior to administration, cells are thawed, washed and reconstituted at the clinical site, as detailed below.

### Study endpoints

#### Primary endpoints: safety

Because this is a first-in-humans application of human MSCs in patients with moderate-severe ARDS, the primary study endpoints for both phase 1 and phase 2 will focus on the safety and tolerability of the human MSCs product. In phase 1, this analysis will examine (1) the incidence of pre-specified infusion associated events and (2) unexpected severe adverse events in ARDS patients treated with human MSCs. For phase 2, the MTD will be used, and the primary endpoint will be (1) the incidence of pre-specified infusion associated events and (2) the rate of unexpected severe adverse events observed in ARDS patients treated with human MSCs compared to patients treated with placebo.

Because infusion of MSCs could theoretically cause transient obstruction of the pulmonary microcirculation leading to (1) a fall in systemic blood pressure, (2) an increase in vasopressor dose, (3) an increase in heart rate, (4) an increase in arterial carbon dioxide concentration, or a (5) a decline in oxygenation, patients will be monitored closely by a study physician during the infusion and for six full hours following the start of the infusion for changes in any of these parameters. Pre-specified infusion associated events will be defined as one of the following events that occurs within six hours of the MSCs infusion: an increase in vasopressor dose greater than or equal to a predefined threshold, new ventricular tachycardia, ventricular fibrillation or asystole, new cardiac arrhythmia requiring cardioversion, hypoxemia requiring an increase in the fraction of inspired oxygen of 0.2 or more and an increase in the level of PEEP of 5 cmH_2_0 or more to maintain transcutaneous oxygen saturations in the target range of 88 to 95%, or a clinical scenario consistent with transfusion incompatibility or transfusion-related infection. A two-hour stability period will be required prior to MSC infusion. In addition, cardiac arrest/death within 24 hours of infusion would be considered a pre-specified infusion associated event, since patients who are moribund and not expected to survive 24 hours will be excluded from the trial (Table 1).

**Table 1:** Pre-specified infusion associated adverse events for START

**Within six hours of the MSCs infusion**	**Within 24 hours of the MSCs infusion**^**b**^
1. An increase in vasopressor dose greater than or equal to a predefined threshold:	1. Cardiac arrest
> 10 mcg/minute norepinephrine	2. Death
> 100 mcg/minute phenylephrine	
> 10 mcg/kg/minute dopamine	
> 0.1 mcg/kg/minute of epinephrine^a^	
Addition of a third vasopressor	
2. New ventricular tachycardia, ventricular fibrillation or asystole	
3. New cardiac arrhythmia requiring cardioversion	
4. Hypoxemia requiring an increase in the fraction of inspired oxygen of 0.2 or more and an increase in the level of PEEP of 5 cmH_2_0 or more to maintain transcutaneous oxygen saturations in the target range of 88 to 95%	
5. A clinical scenario consistent with transfusion incompatibility or transfusion-related infection (urticaria, wheezing)	

^a^Although patients on epinephrine were excluded from the original clinical trial protocol, because of the current Surviving Sepsis Guidelines and the use of epinephrine as a first line vasopressor in at least one study hospital ICU, the protocol was amended to allow patients on a modest dose of epinephrine to be included in the trial and to define a pre-specified infusion associated event based on epinephrine dosing.

^b^Since patients who are moribund and not expected to survive 24 hours are excluded from the study.

The predefined threshold for increased vasopressor dosing qualifying as a pre-specified infusion-associated adverse event are described as in Table 1: an increase of more than 10 mcg/minute norepinephrine, 100 mcg/minute phenylephrine, 10 mcg/kg/minute dopamine, 0.1 mcg/kg/minute of epinephrine or addition of a third vasopressor. These predefined thresholds were determined based on what investigators would consider significant increases in vasopressor dosing.

In addition to these pre-specified infusion associated adverse events, we will also systemically collect and review the incidence and nature of all serious adverse events for the duration of the clinical trial, with special attention to adverse events that are unexpected in the clinical course of a critically ill patient with ARDS.

#### Secondary endpoints: potential indicators of treatment efficacy

Given the proposed paracrine effects of human MSCs, we will test three categories of efficacy endpoints in the phase 2 trial: respiratory, systemic and biologic. Respiratory efficacy endpoints will include the Lung Injury Score (LIS) at day 3, since improvement in the LIS has been shown to be associated with other clinical outcomes [5],[20],[21], including an increased number of ventilator free days and improved survival. The LIS is a composite scoring system including the PaO_2_/FiO_2_, the level of positive end-expiratory pressure, the extent of infiltrates on the chest radiograph, and static respiratory compliance. The other respiratory efficacy endpoints will include the PaO_2_/FiO_2_ ratio and oxygenation index (OI) at day 3, which incorporates mean airway pressure and the PaO_2_/FiO_2_. OI is independently predictive of mortality in patients with ARDS [22],[23].

Systemic efficacy endpoints will include the mean Sequential Organ Failure Assessment (SOFA) score [24] at day 3 as well as ventilator-free [25], ICU-free, vasopressor-free, and organ failure free days and 60 day all-cause mortality, noting that this initial phase 2 clinical trial of 60 patients will be underpowered for these endpoints.

Biological endpoints will focus on the proposed mechanisms of action of the human MSCs in ARDS based on preclinical studies. Specifically, we will measure plasma markers of lung epithelial injury (Receptor for Advanced Glycation Endproducts (RAGE)), pro- and anti-inflammatory markers (IL-6, IL-8, IL-10 and IL-1Ra), markers of endothelial injury (von Willebrand factor, angiopoeitin-2), markers of other organ injury (creatinine) and biomarkers that may reflect the paracrine activity of the administered human MSCs (angiopoeitin-1 and keratinocyte growth factor). All of these biomarkers will be measured at baseline and day 3 using enzyme-linked immunoassays (ELISAs). In the phase 2 study, we plan to measure a marker of lung epithelial permeability (total protein concentration in a mini-bronchoalveolar lavage obtained 48 hours after product infusion using a colorimetric assay) [26] as well as the total and differential cell count in the mini-bronchoalveolar lavage specimen.

### Selection of study subjects

The inclusion and exclusion criteria (Table 2) are intended to identify a group of patients with sufficiently high mortality to benefit from MSC therapy, but also to exclude patients at higher risk of complications since this is a phase 1/2 trial. The inclusion criterion for study entry is the presence of moderate-severe ARDS, defined as the acute onset of the need for positive pressure ventilation by an endotracheal or tracheal tube with a PaO_2_/FiO_2_ ratio < 200 with at least 8 cmH_2_O positive end-expiratory airway pressure (PEEP), bilateral infiltrates consistent with pulmonary edema on frontal chest radiograph and no clinical evidence of left atrial hypertension for the bilateral pulmonary infiltrates. To avoid enrolling patients with late ARDS, patients must be enrolled and randomized within 96 hours of meeting the Berlin definition for ARDS (Table 2, exclusion 2). At the time of randomization, all inclusion criteria must be met. Subjects must receive the study infusion (either MSCs in phase 1 or MSCs/placebo in phase 2) within 120 hours of meeting the Berlin definition for ARDS. During this period, the PaO_2_/FiO_2_ ratio must remain < 300 mmHg on a PEEP of at least 8 cmH_2_O.

**Table 2:** Inclusion and exclusion criteria for START

***Inclusion criteria***	***Exclusion criteria***
Patients will be eligible for inclusion if they meet all of the below criteria. Criteria 1 to 3 must all be present within a 24-hour time period and at the time of enrollment:	1. Age younger than 18 years
1. A need for positive pressure ventilation by an endotracheal or tracheal tube with a PaO_2_/FiO_2_ ratio < 200 with at least 8 cmH_2_O positive end-expiratory airway pressure (PEEP)	2. Greater than 96 hours since first meeting ARDS criteria per the Berlin definition [19]
2. Bilateral infiltrates consistent with pulmonary edema on frontal chest radiograph, and	3. Pregnant or breast-feeding
No clinical evidence of left atrial hypertension, or if measured, a Pulmonary Arterial Occlusion Pressure (PAOP) less than or equal to 18 mmHg	4. Prisoner
	5. Presence of any active malignancy (other than non-melanoma skin cancer) that required treatment within the last two years
	6. Any other irreversible disease or condition for which six-month mortality is estimated to be greater than 50%
	7. Moderate to severe liver failure (Childs-Pugh Score > 12)
	8. Severe chronic respiratory disease with a PaCO_2_ > 50 mmHg or the use of home oxygen
	9. Patient, surrogate, or physician not committed to full support (exception: a patient will not be excluded if he/she would receive all supportive care except for attempts at resuscitation from cardiac arrest).
	10. Major trauma in the prior five days
	11. Lung transplant patient
	12. No consent/inability to obtain consent
	13. Moribund patient not expected to survive 24 hours
	14. WHO Class III or IV pulmonary hypertension [27]
	15. Documented deep venous thrombosis or pulmonary embolism within past three months
	16. No arterial line/no intent to place an arterial line
	17. No intent/unwillingness to follow lung protective ventilation strategy or fluid management protocol
	18. Currently receiving extracorporeal life support or high-frequency oscillatory ventilation

Another unique design feature of this trial is that we have mandated that enrolled patients achieve a two-hour stable baseline period prior to administration of MSCs. The stable baseline criteria are as follows:

Baseline stability criteria that must be met prior to human mesenchymal stem cell (MSC) infusion

In the supine position, patients must sustain:

1. Transcutaneous oxygen saturation in the target range of 88 to 95% without any increase in ventilator settings

2. Stable vasopressor use if the patient requires vasopressors for blood pressure support. The dose of vasopressor may be able to be increased no more than:

 5 mcg/minute for norepinephrine

 50 mcg/minute increase for phenylephrine dose

 5 mcg/kg/minute increase for dopamine dose

 0.5 mcg/kg/minute increase for epinephrine.

These criteria were designed to reduce noise such that a harmful effect of MSCs could be more clearly identified. Cell-based therapy requires coordination with the bone marrow transplantation facility for investigational product preparation. Patients in phase 2 will be randomized after confirmation that the cells can be prepared and delivered to the ICU within four hours and when patients are likely to achieve a two-hour stable baseline period.

### Treatment groups and randomization

In phase 1, there are three planned cohorts with 3 subjects in each cohort who will receive doses of 1 × 10^6^ cells/kg, 5 × 10^6^ cells/kg, and 10 × 10^6^ cells/kg predicted body weight in an escalating fashion. In phase 2, subjects will be randomized in a 2:1 randomization scheme to receive the maximum tolerable dose of MSCs determined in phase 1 or Plasma-lyte A placebo. Sixty patients will be randomized by bone marrow transplant laboratory personnel using a centralized data management system. Blinding of the investigational product will be preserved throughout the study. No treatment group information is provided to the investigators or clinicians caring for the patient except in case of an emergency, and a log of unblinding events will be maintained. An unaffiliated external medical monitor will work with investigators to determine when and if unblinding should occur.

### Study procedures

**Figure 1:**
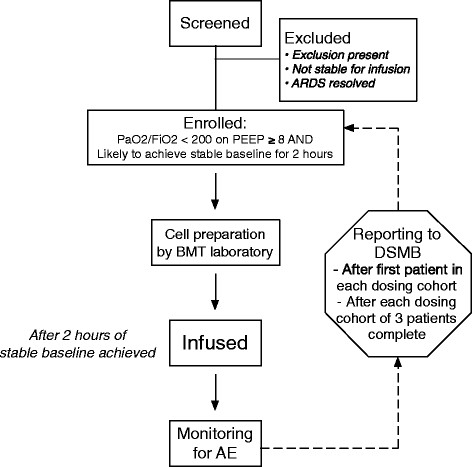
**Flow diagram for the phase 1 component of the START trial.** Abbreviations are as follows: acute respiratory distress syndrome, ARDS; adverse event, AE; bone marrow transplantation, BMT; Data Safety Monitoring Board, DSMB; positive end-expiratory pressure, PEEP.

All study procedures except for pre-screening occur after informed consent is obtained from a subject or his/her surrogate. The study flow is summarized in Figure 1. In phase 1, following informed consent, the bone marrow transplantation laboratory at the clinical site is notified that there is an eligible patient and of the approximate time of the infusion (based on the planned start of the baseline infusion period). The bone marrow transplantation laboratory then determines the dose of cells to be administered based on the patient’s predicted body weight [4]. After initiation of the baseline stability period, the bone marrow transplantation laboratory thaws and washes the human MSCs. The cells are then resuspended in Plasma-lyte A for the study infusion. The final volume of the study infusion is 100 mls (of either human MSCs or placebo Plasma-lyte A).

Because critically ill patients often experience minute-to-minute changes in vital signs, we have mandated that the MSCs infusion can only begin after a two-hour period of stable baseline has been documented. Stable baseline is defined as: transcutaneous oxygen saturation in the target range of 88 to 95% without any increase in ventilator settings and stable vasopressor use if the patient requires vasopressors for blood pressure support. The dose of vasopressor may be able to be increased a small amount during this two-hour period, predefined as in Table 2. If the patient is on vasopressin, investigators will be asked not to titrate the vasopressin dose during this two-hour period. In addition, patients are considered clinically unstable and not eligible to receive MSCs if they require an FiO_2_ ≥ 0.9, PEEP ≥15 cm H_2_O, 3 vasopressors or the use of > 0.1 mcg/kg/minute epinephrine for blood pressure support. Finally, given the recent interest in prone positioning, we amended the protocol to state explicitly that patients must be in the supine position for the baseline period, cell infusion and post-infusion monitoring period to reduce the likelihood of respiratory or hemodynamic instability related to changes in position.

The investigational product can be infused via peripheral or central venous access, though if administered through a peripheral intravenous access, it should be at least 20-gauge, and ideally 18-gauge. The protocol recommends against co-administration with a medication or dextrose-containing solution. The cells are administered through a standard blood filter tubing set with a 170 to 260 micron filter via gravity; droplet count is used to control the infusion rate. The rationale for this approach is that several of the laboratories in our group do not routinely infuse cell-based therapies via an infusion pump, which has the potential to cause mechanical disruption of the cells. The investigational product is infused over approximately 60 minutes. Ventilator management, including weaning, follows the modified ARDS Network lower tidal volume (6 ml/kg predicted body weight) protocol because using this ventilator management protocol will standardize the application of PEEP, which is a component of one of the respiratory efficacy endpoints, the LIS, thus reducing the potential for bias. Patients are managed with a conservative fluid management protocol. The fluid management strategy is held for four hours prior to and six hours after the MSC infusion to reduce the likelihood that the fluid management strategy impacts hemodynamic stability around the time of the study intervention.

Blood and urine samples are obtained at multiple time points before and after the infusion of investigational product for biomarker measurements, which include measurements of epithelial injury, inflammation and of MSC administration. In the phase 2 component, a mini-bronchoalveolar lavage is planned.

For clinical endpoint and safety measurements, if not obtained as part of clinical care, patients will undergo an arterial blood gas measurement and chest radiograph on day 3 (after administration of the investigational product) since these are secondary endpoints of the phase 2 trial. Serum creatinine, total bilirubin and alanine aminotransferase (ALT) are measured on days 3, 7 and 14 (after administration of the investigational product) for safety monitoring if subjects are still hospitalized. Vital status as well as the need for dialysis will be assessed at 6 and 12 months after study enrollment via a telephone visit and in-person visit, respectively. Data on subsequent hospitalizations will also be collected. At the in-person visit, a limited physical exam will be conducted.

### Quality control

At the time of cell thaw at each clinical site, samples of the MSCs are taken by the bone marrow transplantation laboratory for standardized quantitation of cell viability and for cell sterility assays. Protocols for cell thawing and washing as well as infusion were developed at the coordinating center at the University of California, San Francisco. These include the use of standard calculations to determine the appropriate dose of MSCs, standard protocols for cell thaw, transfer and washing, as well as protocols to ensure that the infusion is delivered over the protocol-specified timeframe. These standard operating procedures were adapted at each clinical site and reviewed and approved by the Coordinating Center prior to initiation of study enrollment at each site. Given the cell-based nature of this therapy and concerns about cell viability with variability in cell handling, this is a critical step for the implementation of a cell-based therapy study.

### Study variables

Detailed demographic data and medical history, including smoking and alcohol history and baseline renal function, as well as data for baseline severity of illness, including the Acute Physiology, Age, Chronic Health Evaluation (APACHE) III [28], SOFA [24], and Brussels organ failure scores [29] will be recorded. During the stable baseline, infusion and post-infusion monitoring periods, physiological variables including heart rate, blood pressure, urine output and use of vasopressors, along with respiratory variables including ventilator settings and oxygen saturation will be recorded on a frequent and pre-specified basis. After the investigational product infusion, daily data will be collected for severity of illness scores as well as for lung injury score at days 1, 2, 3, 7, 14, and 28. In addition to clinical data, biospecimens are collected, immediately processed, and stored at −80°C for planned biomarker measurements. All samples will be shipped to the coordinating center at the University of California, San Francisco.

### Sample size

The planned sample size for the phase 1 study is 9 patients (3 cohorts of 3 patients who will receive escalating doses of MSCs using a traditional phase 1 design [30]. The sample size for the phase 2 portion of the trial will be 60 patients randomized 2:1 to receive MSCs or placebo. The sample size was originally based on the proposed primary endpoint of change in four-point acute lung injury score from baseline to day 7 of the study. We selected this endpoint because at least two randomized clinical trials have shown that an improvement in the four-point lung injury score correlates with clinical benefits, including mortality and ventilator-free days, the two most widely accepted clinical endpoints for phase 3 trials in ARDS [5],[20]. With 20 patients in the placebo arm and 40 patients in the MSC arm, we would have 87% power to detect a 0.6 point difference in the lung injury score between the 2 treatment arms, assuming a standard deviation in lung injury score of 1.0, and a correlation of 0.7 between measurements. This effect size is similar to what was reported by Meduri and colleagues for a methyprednisolone infusion for early ARDS (0.54 point change in lung injury score over 7 days, *P* = 0.004) and smaller than that reported for higher dose methylprednisolone for late ARDS (1.3 point change, *P* = 0.001) [31],[32]. However, during the final design of the phase 2 trial, we revised the primary endpoint of the study to focus on safety, rather than efficacy. At the current sample size, as detailed in the Analysis plan, the trial will allow for estimates of the proportion of subjects who experience an adverse event in the MSC and placebo-treated arms as well as the standard effect size of MSCs for clinical outcomes. This clinical and biological data will aid in sample size projections for subsequent phase 2b/3 clinical trials.

## Results and discussion

### Analysis plan

For the phase 1 study, the analysis will be descriptive. Specifically, we will describe the incidence of serious adverse events, including death, as well as the incidence of pre-specified infusion associated events and non-serious adverse events felt to be related to the study infusion.

For the phase 2 study, we will describe the incidence of serious adverse events, including death, as well as the incidence of pre-specified infusion associated events and non-serious adverse events felt to be related to the study infusion in the MSC-treated versus placebo arms. Analysis of the primary safety endpoint will be focused on characterizing the adverse event proportion in each treatment arm, whereas the secondary efficacy endpoints will be used for the design of larger efficacy studies. We will compare the incidence of adverse events by treatment cohort using the Pearson’s chi-square test or the Student’s *t*-test. The per-treatment arm sample sizes were generated based on an assumption of a 28-day adverse event proportion of 30%. In this setting, the 95% CI length for a binomial proportion is 24%, ranging from 19% to 43%.

Because the phase 2 trial is focused on safety and has limited power for physiological endpoints, the analysis will be based on an approach using standardized effect sizes (the difference in mean values between treatment and control divided by the standard deviation). This approach allows us to evaluate the efficacy of MSCs in a small sample size by comparing the effect size observed in our phase 2 trial to effects observed in larger trials of therapies known to be efficacious. In the case of ARDS, the comparison trials would be those of lower tidal volume ventilation [4] and fluid conservative therapy [5]. We will use the incidence of adverse events along with an overall assessment of the potential efficacy of MSCs using the pre-specified respiratory, clinical and biological endpoints to make a determination as to whether or not a larger phase 2b study is safe and warranted.

### Data safety and monitoring

As described in the prior sections, unique features of this clinical trial include the requirement for a two-hour baseline stability period prior to investigational product infusion and the definition of pre-specified infusion associated events. These are intended to ensure subject safety despite underlying critical illness and to minimize the potential for instability due to the severity of underlying illness rather than the investigational product.

The data safety and monitoring plan for this phase 1/2 trial includes a formal Data Safety Monitoring Board (DSMB) that has been reviewed and approved by the IRB at the University of California, San Francisco and the National Heart, Lung and Blood Institute. The DSMB includes critical care physicians with phase 1/2 trial experience and a biostatistician. The DSMB is required to provide recommendations about starting, continuing, and stopping the study. In addition, the DSMB is asked to make recommendations, as appropriate, to the NHLBI about: benefit/risk ratio of procedures and participant burden; selection, recruitment, and retention of participants; adherence to protocol requirements; completeness, quality, and analysis of measurements; amendments to the study protocol and consent forms; performance of individual centers and core labs; participant safety; notification of and referral for adverse events.

Since this is a phase 1/2 clinical trial focused on safety and since the patient population is critically ill and at high risk for complications related to their underlying severity of illness, we have also appointed a designated external medical monitor and constituted a scientific review committee, chaired by the external medical monitor. In phase 1, the scientific review committee plays a critical role in evaluation of each patient in each dosing cohort to ensure patient safety. Specifically, in phase 1, the first subject in each cohort who receives the MSC infusion is observed for seven days prior to enrollment of the remaining subjects in that cohort. A report of clinical data and adverse events from the first subject in the cohort is reviewed by the scientific review committee. If the patient meets stopping criteria (pre-specified infusion associated event or serious adverse event including death within seven days) or there is concern on the part of the scientific review committee, enrollment will be suspended pending review by the DSMB. If stopping criteria are not met, and there are no safety concerns, the next two subjects in that cohort may be enrolled. The second and third subjects in the cohort may be enrolled concurrently; however if the second subject experiences a pre-specified clinically important event or unexpected serious adverse event, including death, prior to enrollment/dosing of the third study subject, enrollment/dosing will be suspended pending review by the DSMB. After completion of enrollment of each study cohort and seven days of follow-up for all three individuals in the cohort, an aggregate report of clinical data and all adverse events will be reviewed by the scientific review committee and DSMB for each cohort. After completion of the phase 1 study (28 days of follow-up for all study subjects), the scientific review committee will review the data and propose a cell product dose for the phase 2 study. This recommendation will be submitted to the DSMB for approval prior to initiating the phase 2 study.

In phase 2, the external medical monitor will assist the study with adjudication of severe adverse events on a case-by-case basis.

## Conclusions

Phase 1/2 trials of a cell-based therapy in critically ill subjects pose unique design challenges, including the need for clearly defined criteria for clinical stability prior to study treatment and the need to define infusion associated adverse events in order to try to separate medical events related to the patient’s overall condition from events that could be related to the cell-based therapy. The need to define ‘baseline stability’ poses additional challenges related to the need to coordinate cell administration with the bone marrow transplantation laboratory. We describe here some of our proposed solutions to these issues, which should be applicable to both cell-based therapy trials for other diseases, as well as most other early phase trials based in the ICU.

## Abbreviations

ARDS: Acute respiratory distress syndrome

(APACHE) III: Acute Physiology, Age, Chronic Health Evaluation

DSMB: Data safety monitoring board

LIS: Lung injury score

MTD: Maximally tolerated dose

MSC: Mesenchymal stem cells

OI: Oxygenation index

PEEP: Positive end-expiratory pressure

SOFA: Sequential Organ Failure Assessment

## Competing interests

None of the authors has any competing (financial or non-financial) interests to declare.

## Authors’ contributions

KDL and MAM conceived of the study, and participated in its design and coordination and helped to draft the manuscript. HZ was responsible for study coordination throughout the design and implementation phases. LC, MM, DM and AL were responsible for study design and implementation relevant to MSC harvest and well as processing in the bone marrow transplantation lab. XF was responsible for the design and implementation of potency assays for the trial. KC assisted with design and rollout of the study at subsites. JWL was involved in the design of the cell wash protocol. JGW, CSC, JG, AR, JL, KNK, JW-K and BTT were involved in study design. KLD assisted with the statistical analysis plan. All authors read and approved the final manuscript.
